# The Barber Surgeons

**Published:** 1912-12

**Authors:** George Parker

**Affiliations:** Physician to the Bristol General Hospital; Lecturer in Forensic Medicine in the University of Bristol


### THE BARBER SURGEONS.

BY

George Parker, M.D., M.A. Cantab.,

*Physician to the Bristol General Hospital;*
*Lecturer in Forensic Medicine in the University of Bristol.*

A curious order of Minor Surgeons was found in Europe in the Middle Ages, of which perhaps the last survivors are the Feldschers of Russia. Their importance in the history of medicine, as possessing the chief medical organisation of the time, is greater than appears on the surface. They combined surgery with the art of the barber, and the combination once made survived through all vicissitudes down to the middle of the eighteenth century. The origin of the strange union has been sought by various writers in (1) certain customs of ancient Rome; (2) the work of lay servants attached to monasteries, since many of the monks were bled and shaven at regular intervals; (3) in the decree of the Council of Claremont, which forbad priests to act as surgeons; and (4) in societies of the keepers of public baths. It seems that a great fashion for baths arose during the leprosy epidemics in Europe, and reached its height during the thirteenth and fourteenth centuries. The bath-keepers, not content with shaving, dressing and perfuming their clients, treated fractures and dislocations, cupped and bled them, and did simple dentistry. These Barber Surgeons, according to Baas, appeared in Germany as early as 1150, and were for a time the only medical practitioners to be found. Baas notes, too, a curious survival of this union of the two professions in the custom of military surgeons shaving their men, which they continued to do down to the eighteenth century, while they are still called Feldscherers up to the present day in Germany. In France the surgeons were in theory separated from the barbers by a Statute of 1428, and indeed the highest class of them had abjured "barbery," and had been formed into the College of St. Como, under Pitard, as early as 1250. The order of Minor Surgeons, or men of the short robe, remained, however, to a late period. We must, however, pass over the details of their history on the continent.

In Great Britain we find them organized as Craft Guilds in most large towns before 1300, and, indeed, at the Inquisition of 1386, under Richard II, certain of them are returned as existing from unknown antiquity. Now these Livery Companies of Barber Surgeons, as they are termed in later times, are of interest for three reasons:—

(1) Their legal powers were so great, that not only Barber Surgeons, but every rank and kind of surgeon found it useful to join them, and their history for some four centuries is practically that of all the surgeons in the country, though this was a period of great advance in surgery. In some places physicians joined the Company also, and in Edinburgh it at one time included apothecaries. A Company might thus be a Federation or union of all medical interests in its district, with ample powers to protect them. Sometimes the surgeons and barbers in a Company formed distinct groups of men, *e.g.* in London after the Act of 1540; sometimes the same individual was allowed to practise both arts. Some Companies contained additional crafts, such as chandlers in Chester, and as time went on, and when membership could be inherited as well as earned by apprenticeship, there were members who followed no craft. The result is to-day that in many Livery Companies not a single member carries on the original trade. In a few instances, Bristol being one, it is possible that the original guild did not at first include surgery at all; but the fashion changed, and in 1532, if not earlier, we find surgeons in the Bristol Company as elsewhere. However, by the middle of the eighteenth century the surgeons changed their minds once more, and insisted on leaving the Companies, and formed societies of their own. Of the old state of things there are few traces left, though the surgeons in Edinburgh, according to Gairdner, still pay a small sum annually to the Barbers, and in London the Barbers Company retains the hall with the paintings and records of the surgeons, while they have handed over to the latter the endowments of the Arris and Gale lectures. In Chester there was lately a single member left still of the Company, but I know of no other survivals, and the Colleges of London, Edinburgh and Dublin replace to some extent the old Companies.

(2) The whole of the surgical education of the country was carried on by them, partly through their system of apprenticeship, and partly through the lectures, examinations, and anatomical demonstrations which the best Companies instituted. The first treatise on anatomy in English, *The Englishman's Treasure*, was written by Vicary, a Master of the London Company.

(3) The right of granting licences to practise surgery, so far as their own towns were concerned, was entirely in their hands, and as the towns were in the habit of granting each other reciprocal rights, licences might be valid for most large towns. This power, after the Act of 1511, was limited by the right of the Bishop to grant licences over the whole of his diocese. The relation of these two licensing bodies is rather obscure, but on the whole they worked harmoniously together in putting down quacks and pretenders. In one town the power of the Company would be derived from a Royal Charter, in another from the Corporation of the town ; but in either the power was amply sufficient to enforce their rules. Still, there was no central board or authority for the whole country, and probably there was great variety in the requirements of the different places.

If we ask what was the state of things in London, we find that a fraternity of consulting surgeons, like those of St. Como, had long been allied with the Company of Barber Surgeons, and finally a special Act of Parliament in 1540 united the two societies. This led to a very active educational body, and one which affected for good many of the provincial Companies. In the London body there was no difference in rank among the surgeons themselves, but they were quite separate from the barbers, though members of the same Company. Indeed, the statute forbade anyone to practise both arts. Whatever class of practice the surgeon aspired to the training was severe. Apprenticed for seven years, he had to attend lectures, anatomy teaching and examinations. After being " ploughed " more or less often like a modern student, he was licensed to practise by the Company, and perhaps by the Bishop as well. The Company insisted on his still attending frequent post-graduate lectures and demonstrations, and on his giving a lecture to his juniors in his turn. Hogarth has left a picture of a dissection in their theatre, and skilled members of the College of Physicians were often engaged as teachers of anatomy.

Four times a year a body was publicly dissected, and private tutorial classes were formed in addition. On Tuesdays a lecture was generally given on surgery or anatomy.

In serious cases the young surgeon was compelled to get a consultation, and if he showed himself inefficient he was liable to be tried and punished.

In London itself the Act of 1540, and the charters of James I and Charles I, giving the power of licensing to the Company clearly followed after, and over-rode the Act of 1511, which made the Bishop the licenser, and it is claimed that the Bishop for a long period only licensed those whom the Company had examined, although the Vicar-General had expert examiners acting as his assessors. The rules of the Company were enforced by the city authorities, who allowed the qualified Barber Surgeon certain exemptions from civic duties in keeping watch and ward, just as we are now freed from serving on juries.

The story of the Barber Surgeons in other towns is varied and curious. As to the Company in York, I am indebted to Dr. G. A. Auden for abstracts of their records. As early as 1318 two Barber Surgeons were paid £6 by the York Corporation for going to heal David le Brus after his wound at Bamburgh. The Ordinances of the Company date from 1400, though they were expanded in 1485 and again in 1592. They were published under the editorship of Dr. Furnivall, but the text does not include the earliest rules or the other records of the Company.

Among various regulations we find that there was to be no trading on Sunday, except bleeding and giving medicines to the sick. Certain officials called Searchers were authorised to forbid the practice of surgery to aliens or new-comers, if after examination they were found incapable, and to this end the patients they treated were to be inspected. Practitioners of medicine as well as surgery appear to have been under the power of the Company. In 1555 they got the London Barber Surgeons to appoint two lecturers on anatomy for them. In 1614 it was agreed that a surgeon should be annually elected as Master in Anatomy to give lectures and two demonstrations a year, as well as to help the Searchers to teach the apprentices at least every six weeks. Every surgeon in turn must give a lecture when called on. In the next year an unfortunate Mr. Francis Brigham "was found insufficient for surgerie," and forbidden by the Corporation of York to practise. The last entry in the Ordinances concerns the apprenticeship of a certain William Brotherton, and is dated in 1781, after which the Company fell into decay.

In Norwich, one of the cities where the return of 1388 described the Company as existing from times to which the memory of man goeth not, the earliest records refer to the religious duties and the altars maintained by the fraternity. Later on, like several other provincial guilds, we find them using the arms of the London Company. Does this mean that the Tudor Charters or the Act of 1540 gave the Londoners any powers over the local bodies, or united them so as to create a national surgical council, like the new College of Physicians? There does not seem any evidence for this view, though some common action may be found among their various licensing bodies.

However, at Norwich in 1561 certain Ordinances of the Physicians and Barber Surgeons declare the union of the existing Norwich guilds, and insist on consultation with an expert in difficult cases. Next, as to education they order that a lecture be given every three weeks. Finally, they seem to restrict practice to those who have served an apprenticeship in the city. As in other cities, the organisation in later days became unpopular, and no more officials were appointed after 1723.[1]

Dublin appears to have had a royal foundation of Barber Surgeons before any other town in the British Isles, for in 1446 Henry VI gave them a charter to "establish the art of chirurgery."

Elizabeth in 1572 issued a new one, in which she referred to her predecessor's gift, and joined to the existing Company another society of surgeons. The combined body was to be called "The Guild of St. Mary Magdalene of Barbers and Chirurgeons." For the next century a flood of political troubles

[1] Williams, *The Barber Surgeons of Norwich.*

and war disturbed the country, but in 1687 a new charter was granted, confirming the authority of the Company over surgeons, barbers, apothecaries, and peruke-makers, and ordering a seven years' apprenticeship. However, the surgical side of the work was a failure, the barbers outnumbered the surgeons by ten to one, and interfered with any reform proposed, so that no check on unskilled practice was possible. Not only did individual surgeons refuse to join or submit to the decaying Company, but in 1721 a new Society of Surgeons appears, who would not join or take over the Royal foundation.

A new Medical School had been formed in 1712, partly by the efforts of Trinity College, and in 1741 there were hardly any surgeons left in the Company, which appealed in vain to the College of Physicians to take them over with all their rights and powers. In 1745 the apothecaries seceded, and in 1784 the Royal College of Surgeons was created to take charge of surgical matters for the whole of Ireland. The old Company was finally dissolved by the Municipal Reform Act of 1840. Its records are now in the library of Trinity College, and include one or more of the Royal Charters. Lengthy extracts are given in Cameron's *History of the Royal College of Surgeons*, including the grant of a coat of arms, which recites "that the Company of Barber Chirurgeons of the Citty of Dublin have for some space used the arms of the Company of the Citty of London with some small difference, being a note of subordination [though having no dependence on any other citty], therefore a new coat is granted, viz. a chevron gules between three cinquefoils azure and a harpe crowned or."

In Edinburgh, too, a powerful Guild of Barber Surgeons had arisen at an early but unknown date, which was established and strengthened by Municipal and Royal Charters. Thus we find in "The seal of cause" granted by the Town Council of Edinburgh to the Crafts of Surgeons and Barbers in 1505,[1]

[1] "*A Collection of Royal Grants and other Documents Relative to the Constitution and Privileges of the Royal College of Surgeons of Edinburgh from* 1505 *to* 1813. I owe to Dr. James Young much of the story of the Edinburgh Company. Gairdner has also given many details in the *Edinburgh Medical Journal*, 1859 and 1860, but the chief records seem still to be unpublished.

and confirmed by a charter of King James IV in their favour a year later, various rules as to medical education and practice. There is one clause, however, which does not come under this head, but which, as showing the curious mixture of occupations at the time, is too interesting to be omitted. This clause lays down that " no person may make or sell *aqua vitæ* in this burgh except he be a freeman of this craft." How the Edinburgh surgeons lost this monopoly of the sale of whisky is a question I cannot answer. There would be no need of the Carnegie fund if the society had retained it to the present day. Among the other privileges, rules and statutes, it was granted that they should have " once a year one condemned man after he be dead to make anatomy of, wherethrough we may have experience each one to instruct others, and we shall do suffrage for his soul." Again, it was laid down that no person shall occupy or use any points in our said crafts within this burgh unless he be first freeman and burgess of the same, and that he be worthy and expert in all the points belonging to the said craft, having been diligently examined and admitted by the masters of the said craft. Especially he must be examined and tested in anatomy, in the nature and complexion of every member of man's body, and likewise in all the veins of the same. No barber of this burgh shall practise surgery unless he be expert, and know perfectly the things above written. No one shall be taken as apprentice or paid servant until he can read and write. The fine for admission as freeman was five pounds usual money of Scotland, which was employed for the support of the guild altar of St. Mungo. One legitimate son of every freeman was exempt from the payment of the fine, when after the usual examination he was admitted to the craft.

Exemption from bearing arms, serving on inquests and assizes (? juries), was granted to them by Queen Mary in 1567, provided that they paid their rates and gave medical aid to the armies in time of war.

The similarity between these rules and those of the English societies is at once apparent. Confirmation was given by a new charter in 1613, and finally by an Act of Parliament in 1641, which gave their officers power to arrest and fine all persons practising surgery who were not members of the guild. To show that by surgery they did not mean merely blood-letting and tooth-drawing, another Act two years later defines it as "operations and applications upon the living and dead bodies of men, women and children, and the curing of diseases such as tumours, wounds, ulcers, luxations and fractures, the curing of verolls, etc." This Act also went on to lay down various penalties on any apothecary or other person who should carry out surgical treatment if not a member of the guild. A union of the apothecaries and surgeons was attempted rather later on, and then in the eighteenth century the usual break-up of the guild companies took place here as elsewhere, and the Royal College of Surgeons was founded by charter in 1778.

In Bristol the history of the Company is much the same, and the organisation in fashion in London and elsewhere at a given time was carefully copied here.

I have given many details in a paper in this *Journal* for September, 1911 ("Medical Organisation, etc., in the Seventeenth Century"), and I shall only refer to the chief facts here. The scantiness of the Bristol records is partly due to a fire which consumed the collection of Mr. Thomas Kerslake, bookseller, of Park Street, partly to the riots of 1833, and partly to the neglect into which the old guilds fell.

While a great advance in the organisation of Barber Surgeons took place in London, York, and Norwich, towards the end of the fourteenth century, their members being then probably increased by the field surgeons from the French wars, there is no evidence that in Bristol at that date the Company included surgeons at all. At any rate *The Little Red Book*, a collection of the Ordinances of the time, says nothing as to surgery or the teaching of anatomy, but regulates the barbers of the Company in 1395, 1418, and 1439. The Company is mentioned among those which were entitled to share the municipal feasts (28 Henry VI), and it generally took precedence as third among the 23 Livery Companies.

The inclusion of surgery or surgeons, if not original seems to have been effected before 1532, for in August of that year one Thomas Rogers on receiving the freedom of the city is described as a Barber Surgeon.[1] Fresh Ordinances were granted from time to time, and when, in 1652, the existing ones had become partly illegible, the ancient "Company of Barbers and Chirurgians" once more obtained new ones from the City Council. A copy of this charter is in the possession of Mr. Francis Fox of Yate House, and gives a clear picture of their work. Thus apprenticeship for seven years was insisted on. No one might practise surgery unless (1) he had been passed by the Company as skilful in the art, and (2) he had been made free, *i.e.* a member of the Company. Heavy fines were incurred by neglect of a patient or malpractice, and a consultation was insisted upon in serious cases, or what we should term major operations. Surgical aid for the poor was to be provided gratis by the Company when necessary, and the surgeon's chest on any ship leaving the port was to be examined by the Company's officer.

The Bishop's Vicar-General in 1670 claimed that their licensees must take out his licence as well under the Act of 1511, but the City Council supported them so firmly that the Bishop's claim was at once dropped. As to other educational matters there is little information extant, for the minute books and most of the Ordinances are lost, and not a single document from 1450 to 1650—the period of the greatest activities of the Companies—has come down to us. However, it is clear there were rules penalising professional ignorance, and their Surgeons' Hall and Anatomical Theatre are still in existence. It is significant, too, that no sooner had the Company broken up, about 1742, than the Surgeons of the Royal Infirmary instituted lectures on anatomy, apparently to replace those of the Company, and these gradually led up to the Bristol Medical School.

In the eighteenth century a general dislike of the trade guilds grew up as devices in restraint of trade, and utterly opposed to the *laissez-faire* system. This view is most ably expressed by Adam Smith. Whatever the cause, the Bristol Company

[1] The Chamberlain's Accounts, Bristol.

like others fell quickly into decay. The better surgeons refused to take out its licence, though how they escaped prosecution is not clear. The surgeons' work, too, was largely engrossed by the apothecaries. Within the Company violent disputes between the Surgeons and the Barbers can be traced by their petitions to the City Council. In 1739 Isaac Page and 31 of the Barbers complained of "the other part of the Company called Surgeons." The master and wardens then appeal about these "uneasie members," and another petition in 1740 speaks of the great inconvenience of the present ordinances and prays for their revision.[1]

At the opening of the Exchange in 1742 the Surgeons' Company, as it was then called, for the last time joined in the mayor's procession, "marching with music." They had recently erected for themselves a fine new hall, a large part of which still remains as the Grapes Hotel and the Grotto Restaurant. But even the Barbers deserted the Company, and the disused hall had to be let. The few remaining members can be traced mortgaging it in 1750, and finally selling it to Matthew Wright in 1790, after which they disappeared altogether.[2]

In Glasgow, Newcastle-on-Tyne, Cork, Limerick, and probably many other ancient towns, there were similar other Companies, but no details are to hand with respect to them. In Oxford a Company was formed in 1348, and continued till as late as 1850, but no trace of one has been found at Cambridge.

In Chester the Company was created by the City Corporation, and ranked third on the list of the twenty-three guilds. A charter of 1540 refers to the seven years' apprenticeship, but the period seems really to have varied from four to twelve years. With the Barber Surgeons there was united in one Company a body of chandlers, a form of amalgamation which seems to have been popular in Chester, where the apothecaries

[1] Minute Books of the City Council, May 16th, 1739; March 22nd, 1740.

[2] Braikenridge Collection, All Saints' volume, p. 419. Haberfield gives the names and other details of these transactions.

The City Burgess Rolls contain names of all Barber Surgeons from 1598.

ironmongers, grocers, and mercers formed another guild. In Mr. Simpson's abstract of their rules there is nothing laid down as to consultations, medical education or examinations. Though the Company seems to have claimed relationship with the great London Society by using their arms, there is nothing to show that they imitated their zeal for the advance of surgery. However, the records are not published *in extenso*, and their good deeds may be hidden.

To sum up these local conditions. We find everywhere, after a certain date, that the government of various trades and professions was entrusted to certain craft guilds, and one of these guilds in most towns regulated surgical education and practice. Very often this guild contained barbers and surgeons, or individuals practising both arts ; sometimes it included other interests. After this system had lasted 300 or 400 years, economic changes, such as the introduction of the factory system, led to such general dislike of the guilds, that they became discredited and withered away. Among them the surgical guild societies perished in the first half of the eighteenth century, and were replaced in some instances by Royal Colleges, who took over the disciplinary and educational work of the Companies, and sometimes even their endowments. This involved to some extent the substitution of a national licensing body for numerous local ones, and so far it was a gain ; but how far the educational and disciplinary work they undertook is a success, and whether it satisfactorily replaces the work of the Companies in their palmy days, is another matter.

